# Design and simulation investigations on charge transport layers-free in lead-free three absorber layer all-perovskite solar cells

**DOI:** 10.1007/s12200-024-00119-1

**Published:** 2024-06-11

**Authors:** Guangdong Li, Mingxiang Xu, Zhong Chen

**Affiliations:** School of Physics, Southeast University, Nanjing, 211189 China

**Keywords:** Multiple absorber layer PSCs, CTLS-free, Inverted, Narrow-band gap, Simulation

## Abstract

The multiple absorber layer perovskite solar cells (PSCs) with charge transport layers-free (CTLs-free) have drawn widespread research interest due to their simplified architecture and promising photoelectric characteristics. Under the circumstances, the novel design of CTLs-free inversion PSCs with stable and nontoxic three absorber layers (triple Cs_3_Bi_2_I_9_, single MASnI_3_, double Cs_2_TiBr_6_) as optical-harvester has been numerically simulated by utilizing wxAMPS simulation software and achieved high power conversion efficiency (PCE) of 14.8834%. This is owing to the innovative architecture of PSCs favors efficient transport and extraction of more holes and the slender band gap MASnI_3_ extends the absorption spectrum to the near-infrared periphery compared with the two absorber layers architecture of PSCs. Moreover, the performance of the device with p-type-Cs_3_Bi_2_I_9_/p-type-MASnI_3_/n-type-Cs_2_TiBr_6_ architecture is superior to the one with the p-type-Cs_3_Bi_2_I_9_/n-type-MASnI_3_/n-type-Cs_2_TiBr_6_ architecture due to less carrier recombination and higher carrier life time inside the absorber layers. The simulation results reveal that Cs_2_TiF_6_ double perovskite material stands out as the best alternative. Additionally, an excellent PCE of 21.4530% can be obtained with the thicker MASnI_3_ absorber layer thickness (0.4 µm). Lastly, the highest-performance photovoltaic devices (28.6193%) can be created with the optimized perovskite doping density of around E15 cm^3^ (Cs_3_Bi_2_I_9_), E18 cm^3^ (MASnI_3_), and 1.5E19 cm^3^ (Cs_2_TiBr_6_). This work manifests that the proposed CTLs-free PSCs with multi-absorber layers shall be a relevant reference for forward applications in electro-optical and optoelectronic devices.

**Graphical Abstract:**

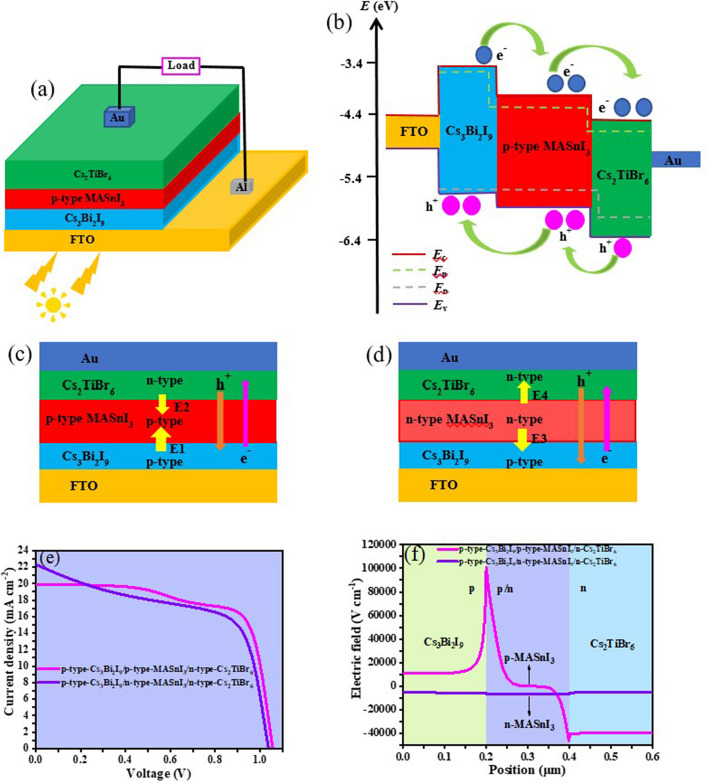

## Introduction

Organic–inorganic haloid lead (Pb)-based perovskite solar cells (PSCs) have drawn enormous regard in the photovoltaic community during the past decades by reason of their simple preparation processing, high optical absorption coefficient, superior charge transport properties and outstanding power conversion efficiency (PCE) [1–5]. The evolutionary PCE of single-heterojunction PSCs has rocketed from the original 3.8% to above 26% [6–9]. However, the chemical instability and toxic nature of Pb spawns critical blackmail to the environment and obstacles the massive commercialization application [10]. To dispose of the issue, substituting Pb with the non-toxic Sn element in the MAPbI_3_ will be a latent light absorption material of the future PSCs. This is owing to the MASnI_3_ material having a narrow band gap which brings about the expanded optical absorption up to 1050 nm [11]. However, Sn^2+^ is easily oxidized to Sn^4+^, resulting in Sn vacancies and high-concentration p-type doping behavior as well as an increased carrier recombination rate [12–14]. Consequently, the performance of the device will be affected to some extent. To improve the performance of the Sn-based PSCs, many researchers are working on multi-absorber layer heterojunction solar cells that can generate superior performance in a longer region of visible spectrum. Farhadi et al. used a novel construction with a double absorbing layer (MASnI_3_/MAPbI_3_) PSCs to achieve 30.88% efficiency [15]. Additionally, Abedini-Ahangarkola et al. proposed three active layer structures (MASnI_3_/MAPbI_3_/FAMASnGeI_3_) and achieved 31.44% high efficiency [16]. However, there are still toxic Pb elements in these perovskite layers.

In this work, an inverted p-p-n planar heterojunction structure PSCs with triple Cs_3_Bi_2_I_9_/single MASnI_3_/double Cs_2_TiBr_6_ three absorber layers are proposed. The upper and lower surfaces of the MASnI_3_ absorber layer can be effectively covered by the Cs_2_TiBr_6_ and Cs_3_Bi_2_I_9_ absorber layers, respectively. Additionally, the triple Cs_3_Bi_2_I_9_ and double Cs_2_TiBr_6_ not only have ideal light absorption properties and non-toxic crises but also have a high level of stability and non-degradability [17–20]. Accordingly, the Cs_3_Bi_2_I_9_ and Cs_2_TiBr_6_ can may be similar to antioxidant additives that diminish the oxidation of Sn^2+^ in the MASnI_3_ absorber layer. In PSCs, the common electron transport layer (ETL) materials are TiO_2_, SnO_2_ and ZnO. Wherein, the preparation of the TiO_2_ layer is usually required at a high post-annealing temperature, which indubitably increases the complicacy of device fabrication [21, 22]. Furthermore, the immediate contact of ZnO, SnO_2_ and the perovskite absorber layer will spawn the degradation of perovskite [23, 24]. In the same way, most used hole transport layer (HTL) materials such as Spiro-OMeTAD and PEDOT: PSS require higher preparation costs and are subject to stability limits [25, 26]. Based on this, to avoid the impacts of these detrimental factors on the efficiency of the PSCs and simplify the preparation process, charge transport layers-free (CTLs-free) PSC devices are gradually being investigated. Furthermore, the PSCs with inversion construction (p-i-n) not only provide superior fixity but also have non-existent evident current density–voltage (*J*–*V*) hysteresis effect [27, 28], which shall be a novel exploration sphere.

Due to the experimental fabricating of the distinct levels of multi-layered PSCs is costly and time-consuming, accordingly, the simulation studies are vital for determining reasonable materials and parameters for different functional layers of PSCs [29]. In our work, we simulated and proposed a novel inverted CTLs-free PSCs structure with triple Cs_3_Bi_2_I_9_/single MASnI_3_/double Cs_2_TiBr_6_ three absorber layers through utilizing wxAMPS simulator and contrasted the photoelectric characteristic with two absorber layers (Cs_3_Bi_2_I_9_/Cs_2_TiBr_6_ and MASnI_3_/Cs_2_TiBr_6_) architecture of PSCs to show the improvement of efficiency in three absorber layer PSCs. We also analyzed and compared of the electrical and photophysical properties of PSCs with different p-p-n and p-n–n heterojunction structures in detail. Moreover, we concentrate on the effects of different double perovskite materials, the thicknesses and doping density of perovskite layers on the photoelectric characteristic of the proposed device at length to assign the optimum material parameters and layered heterojunction architectures. These results can not only pave the path for simplifying the device’s structure and fabricating high-performance CTLs-free PSCs with multi-absorber layers but also allows us to better understand the spatial distribution of internal carriers and charge transport mechanism of the different structure devices.

## Simulation methodology and device configuration

In this work, the numerical simulation of PSCs has been carried out by employing wxAMPS software under the AM 1.5G solar spectrum illumination circumstance and the device operation temperature is taken to be 300 K. The wxAMPS software is available as an updated and optimized version with based on AMPS developed for the numerical simulation of devices by Professor S. Fonash of Pennsylvania State University [30]. The tunnelling effect was contemplated in the wxAMPS mold by incorporating intra-band tunnelling models and trap-assisted tunnelling. The device performance can be analyzed under the simulation program through solving the Poisson equation (Eq. (1)), electrical and hole continuity equations (Eqs. (2) and (3)) [31]. The relational equations are as follows:

1 $$\text{d}(-\varepsilon(x)\text{d}\varphi/\text{d}x)/\text{d}x=q\lbrack p\left(x\right)-n\left(x\right)+N\begin{array}{c}+\\\text{d}\end{array}\left(x\right)-N\begin{array}{c}-\\\text{a}\end{array}\left(x\right)+p_{\mathrm t}\left(x\right)-n_{\mathrm t}(x)\rbrack,$$

2 $$\mathrm{d}p_{\mathrm{n}}/\mathrm{d}_{\mathrm{t}}=G_\mathrm{p}-(p_{\mathrm{n}}-p_{\mathrm{n}0})/\tau_{\mathrm{p}}-p_{\mathrm{n}}\mu_{\mathrm{p}}\mathrm{d}\xi/\mathrm{d}x-\mu_\mathrm{p}\xi\mathrm{d}p_{\mathrm{n}}/\mathrm{d}x+D_{\mathrm{p}}\mathrm{d}^{2}p_\mathrm{n}/\mathrm{d}x^2,$$

3 $$dn_{\mathrm p}/{\text{d}}_{\mathrm t}=G_{\mathrm n}-(n_{\mathrm p}-n_{\mathrm p0})/\tau_{\mathrm n}-n_{\mathrm p}\mu_{\mathrm n}\text{d}\xi/\text{d}x-\mu_{\mathrm n}\xi\text{d}n_{\mathrm p}/\text{d}x+D_{\mathrm n}\text{d}^2n_{\text{p}}/\text{d}x^2,$$

where *ε* represents the permittivity, *φ* represents the electrostatic potential, *q* represents the charge, *p*, *n*, *p*_t_, and *n*_t_ are defined as the densities of free holes, free electrons, trapped holes and trapped electrons, respectively. *N*_d_ and *N*_a_ represent the donor and acceptor doping concentration, *G* represents the generation rate, *ξ* represents the electric field, and *D* represents the diffusion coefficient.

**Fig. 1:**
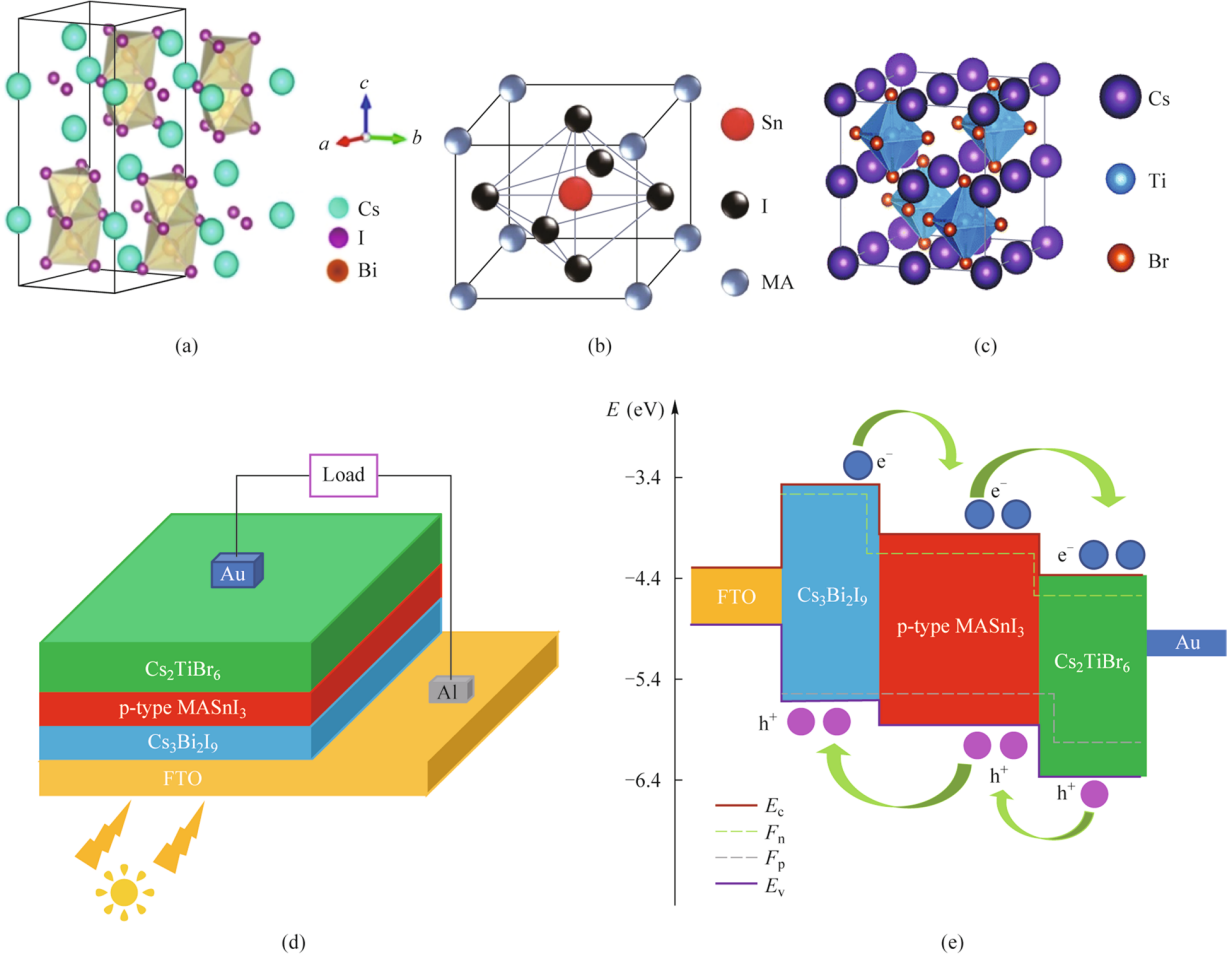
Crystal structure of **a** Cs_3_Bi_2_I_9_, **b** MASnI_3_, and **c** Cs_2_TiBr_6_. **d** Schematic architecture and **e** energy level diagram of the inverted p-p-n heterojunction PSCs

To simplify the architecture of the device and discuss the impact of utilizing multiple absorber layers on the PSC performance, the simulation is depicted on CTLs-free device architecture with an inversion p-p-n planar heterojunction construction comprising the Cs_3_Bi_2_I_9_ absorber layer (Fig. 1a), p-type MASnI_3_ absorber layer (Fig. 1b), Cs_2_TiBr_6_ absorber layer (Fig. 1c), transparent conduction oxide (FTO), and gold (Au) photocathode, as displayed in Fig. 1d. From Fig. 1a − c, the crystal structure of Cs_3_Bi_2_I_9_ shows the hexagonal P6_3_/mmc space group where BiI_6_ octahedra share faces to form [Bi_2_I_9_]^3−^ anions [32]. The MASnI_3_ is a cubic structure and forms a tetragonal symmetry with the P4mm space group at ambience conditions [33]. Additionally, the double perovskite Cs_2_TiBr_6_ has a similar cubic structure with MASnI_3_. Figure 1e displays the energy level diagram of distinct functional layers in the proposed inverted PSC. The differences in electron affinities and band gaps of three different materials give rise to the band offset of the interface. It can be observed that the negative conduction band (*E*_c_) offset of − 0.77 and − 0.28 eV are formed at the interfaces of Cs_3_Bi_2_I_9_/p-type MASnI_3_ and p-type MASnI_3_/Cs_2_TiBr_6_ heterojunction, respectively. Additionally, there is also a negative valence band (*E*_v_) offset of − 0.15 and − 0.42 eV are formed at the interfaces of Cs_3_Bi_2_I_9_/p-type MASnI_3_ and p-type MASnI_3_/Cs_2_TiBr_6_ heterojunction, respectively. Therefore, the negative *E*_c_ offset promotes electron transmission from the Cs_3_Bi_2_I_9_ layer to the photocathode, and the negative *E*_v_ offset can be in favor of hole transmission from the Cs_2_TiBr_6_ layer to the FTO. The materials parameters used in our simulations were summarized from relational literature as seen in Table 1 [11, 17, 19, 34–36]. The electrons and holes thermal velocity are all 10^7^ cm/s. The materials defect states are neutral Gaussian distribution in each functional layer and the defect energy level is situated in the band gap (*E*_g_) center. Additionally, the holes and electrons capture cross sections are all set to 10^−14^ cm^2^ in each functional layer.

**Table 1:** Materials parameters of the theoretical simulated PSCs

Parameters	Cs_3_Bi_2_I_9_	p-type MASnI_3_	n-type MASnI_3_	Cs_2_TiBr_6_
Thickness (µm)	0.2	0.2	0.2	0.2
Relative permittivity *ε*_r_	9.68	8.2	8.2	10
Electron affinity *χ* (eV)	3.4	4.17	4.17	4.47
Band gap *E*_g_ (eV)	2.03	1.41	1.41	1.6
Effective conduction band density *N*_c_ (cm^−3^)	4.98 × 10^20^	1 × 10^18^	1 × 10^18^	1 × 10^19^
Effective valence band density *N*_v_ (cm^−3^)	2.11 × 10^20^	1 × 10^18^	1 × 10^18^	1 × 10^19^
Electron mobility *μ*_n_ (cm^2^/(V⋅s))	4.3	1.6	1.6	44
Hole mobility *μ*_p_ (cm^2^/(V⋅s))	1.7	1.6	1.6	2.5
Acceptor density *N*_A_ (cm^−3^)	1 × 10^11^	1 × 10^17^	0	0
Donor density *N*_D_ (cm^−3^)	0	0	1 × 10^12^	1 × 10^13^
Defect density *N*_t_ (cm^−3^)	2 × 10^15^	2.5 × 10^12^	2.5 × 10^12^	1 × 10^14^

## Results and discussion

### Comparison of Cs_3_Bi_2_I_9_/Cs_2_TiBr_6_, MASnI_3_/Cs_2_TiBr_6_ and Cs_3_Bi_2_I_9_/ MASnI_3_/Cs_2_TiBr_6_ active layer PSCs

**Fig. 2:**
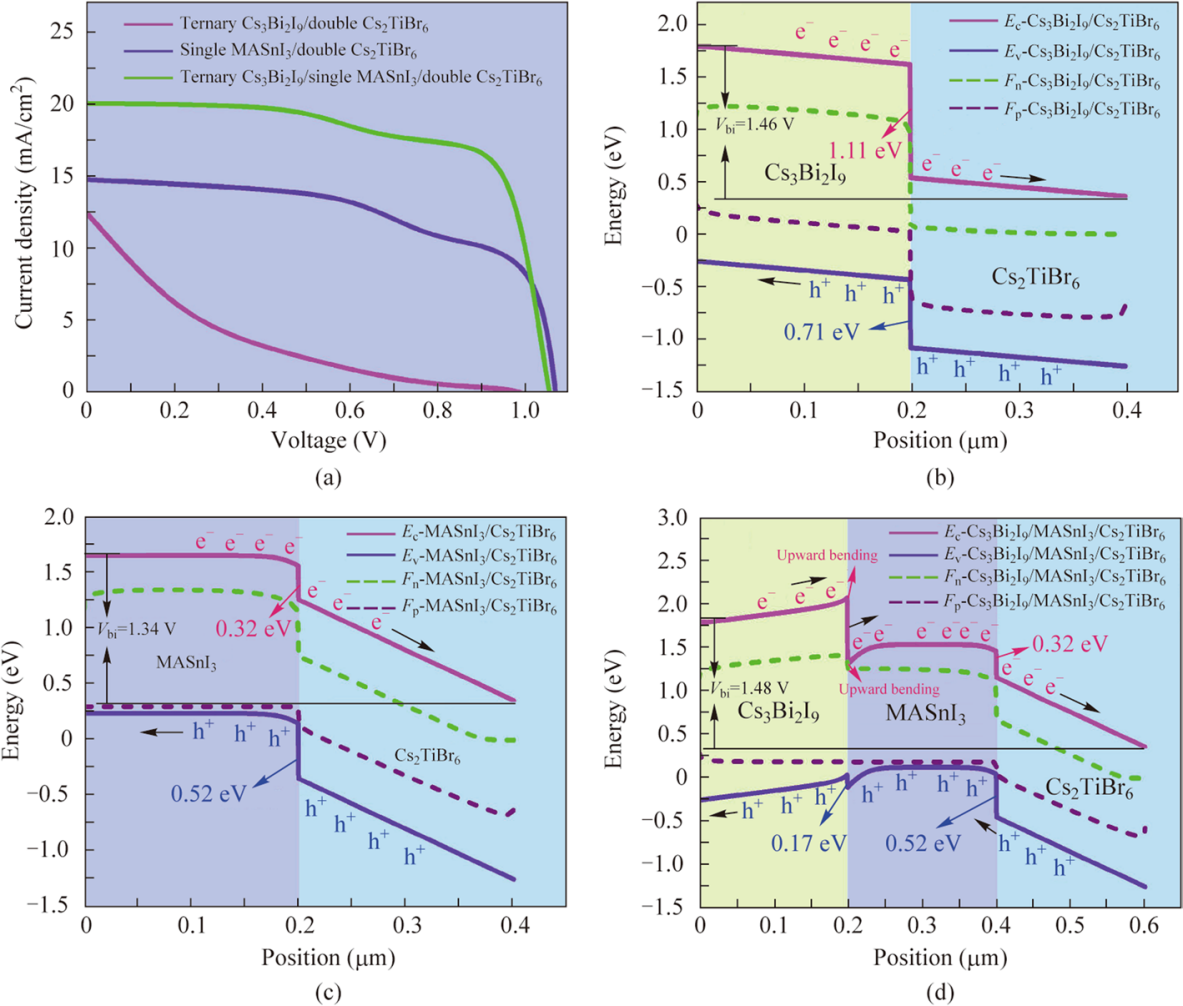
**a** *J* − *V* curves and **b**, **c**, **d** energy band diagrams of the different PSCs with triple Cs_3_Bi_2_I_9_/double Cs_2_TiBr_6_, single MASnI_3_/double Cs_2_TiBr_6_, and triple Cs_3_Bi_2_I_9_/single MASnI_3_/double Cs_2_TiBr_6_ absorber layers

The *J − V* curves of the PSCs with triple Cs_3_Bi_2_I_9_/double Cs_2_TiBr_6_, single MASnI_3_/double Cs_2_TiBr_6,_ and triple Cs_3_Bi_2_I_9_/single MASnI_3_/double Cs_2_TiBr_6_ absorber layers are displayed in Fig. 2a. Table 2 displays the minute device performance parameters derived from our simulation. The detections in Fig. 2a and Table 2 manifest that open-circuit voltage (*V*_oc_) increases from 0.9935 V for Cs_3_Bi_2_I_9_/Cs_2_TiBr_6_ absorber layer PSC to 1.0709 V for MASnI_3_/Cs_2_TiBr_6_ absorber layer PSC. This enhancement in *V*_oc_ can be attributed to the smaller band gap of MASnI_3_ which involves the band offset at the interface of absorber layers and built-in potential (*V*_bi_). Nevertheless, a slight reduction is perceived in *V*_oc_ (1.0569 V) of the Cs_3_Bi_2_I_9_/MASnI_3_/Cs_2_TiBr_6_ absorber layer PSC may be due to the inferior energy barrier at the interface of Cs_3_Bi_2_I_9_/MASnI_3_ absorber layer. Additionally, a remarkable increment of short-circuit current density (*J*_sc_) appears at the Cs_3_Bi_2_I_9_/MASnI_3_/Cs_2_TiBr_6_ (19.9235 mA/cm^2^) and MASnI_3_/Cs_2_TiBr_6_ (14.6741 mA/cm^2^) absorber layer PSCs concerning the Cs_3_Bi_2_I_9_/Cs_2_TiBr_6_ absorber layer counterpart (12.4054 mA/cm^2^). This is owing to the smaller band gap of MASnI_3_ and more absorber layers can facilitate a wider range of photon absorption [16]. Eventually, the highest PCE (14.8834%) of the device was obtained by simulating the PSC of the triple Cs_3_Bi_2_I_9_/single MASnI_3_/double Cs_2_TiBr_6_ configuration. This is attributed to the *J*_sc_ and fill factor (FF) being substantially elevated.

**Table 2:** Performance parameters of the devices with different absorber layers

Device structure	*V*_oc_ (V)	*J*_sc_ (mA/cm^2^)	FF (%)	PCE (%)
FTO/Cs_3_Bi_2_I_9_/Cs_2_TiBr_6_ /Au	0.9935	12.4054	10.7956	1.3305
FTO/MASnI_3_/Cs_2_TiBr_6_ /Au	1.0709	14.6741	58.0687	9.1251
FTO/Cs_3_Bi_2_I_9_/MASnI_3_ /Cs_2_TiBr_6_/Au	1.0569	19.9235	70.6818	14.8834

To further contrast the PSCs’ characteristics of these three configurations, Fig. 2b − d show the energy band diagrams of the proposed PSCs configuration with different absorber layers. As can be seen in Fig. 2b − d, the built-in potentials which are defined by the energy level difference of the *E*_c_ at the beginning of the absorber layer and the end of the absorber layer, are 1.46, 1.34, and 1.48 V for Cs_3_Bi_2_I_9_/Cs_2_TiBr_6_, MASnI_3_/Cs_2_TiBr_6_, and Cs_3_Bi_2_I_9_/MASnI_3_/Cs_2_TiBr_6_ absorber layer PSCs, respectively. The larger *V*_bi_ can effectively facilitate the separation and migration of carriers in the device, which will be more conducive to higher open circuit voltage [37]. However, this *V*_bi_ value is inconsistent with Table 2 which presents that the minimum and maximum *V*_oc_ (0.9935 and 1.0709 V) corresponding to the Cs_3_Bi_2_I_9_/Cs_2_TiBr_6_ and MASnI_3_/Cs_2_TiBr_6_ absorber layer PSCs, respectively. This is attributed to the overlarge band offset at the *E*_c_ and *E*_v_ interface of Cs_3_Bi_2_I_9_ and Cs_2_TiBr_6_ which is adverse to the transport of charge carriers in Fig. 2b. Besides, this large band offset perchance brings about the probability of more carrier recombination. Consequently, overlarge band offset and inferior carrier transport can seriously affect the *V*_oc_ and PCE of the device even if an ideal value of the *V*_bi_ in this device. As can be seen from Fig. 2c and d, two lesser band offsets of 0.32 and 0.52 eV are bespoke at the *E*_c_ and *E*_v_ interface of MASnI_3_ and Cs_2_TiBr_6_, respectively. The lesser band offset will be favorable for the better separation and transmission of charge carriers from the perovskite layer to the electrode. However, In Fig. 2d, the Cs_3_Bi_2_I_9_-MASnI_3_ junction eventuates the energy band upward bent. The upward bending of the energy band not only hinders the transmission of electrons from the Cs_3_Bi_2_I_9_ absorber layer to the MASnI_3_ absorber layer but also prompts more recombination of holes and electrons inside the Cs_3_Bi_2_I_9_ absorber layer and near the interface of Cs_3_Bi_2_I_9_/MASnI_3_. As a consequence, the reason for the *V*_oc_ (1.0569 V) of the Cs_3_Bi_2_I_9_/MASnI_3_/Cs_2_TiBr_6_ absorber layer PSCs is marginally lower than that of the MASnI_3_/Cs_2_TiBr_6_ absorber layer PSCs (1.0709 V) can be explained the *V*_oc_ of the device is affected by the carrier recombination. Additionally, it is worth mentioning that the three absorber layers of perovskite are more conducive to the transport and extraction of more holes from the valence band to the electrode and the hole quasi-Fermi energy level (*F*_p_) is approach to the valence band, as depicted by the valence band curve in Fig. 2d. There is no doubt that the *J*_sc_ of the Cs_3_Bi_2_I_9_/MASnI_3_/Cs_2_TiBr_6_ absorber layer PSCs can be prominently augmented. According to the above analysis, the PSCs configuration with three absorber layers shows the best *J*_sc_ and PCE.

**Fig. 3:**
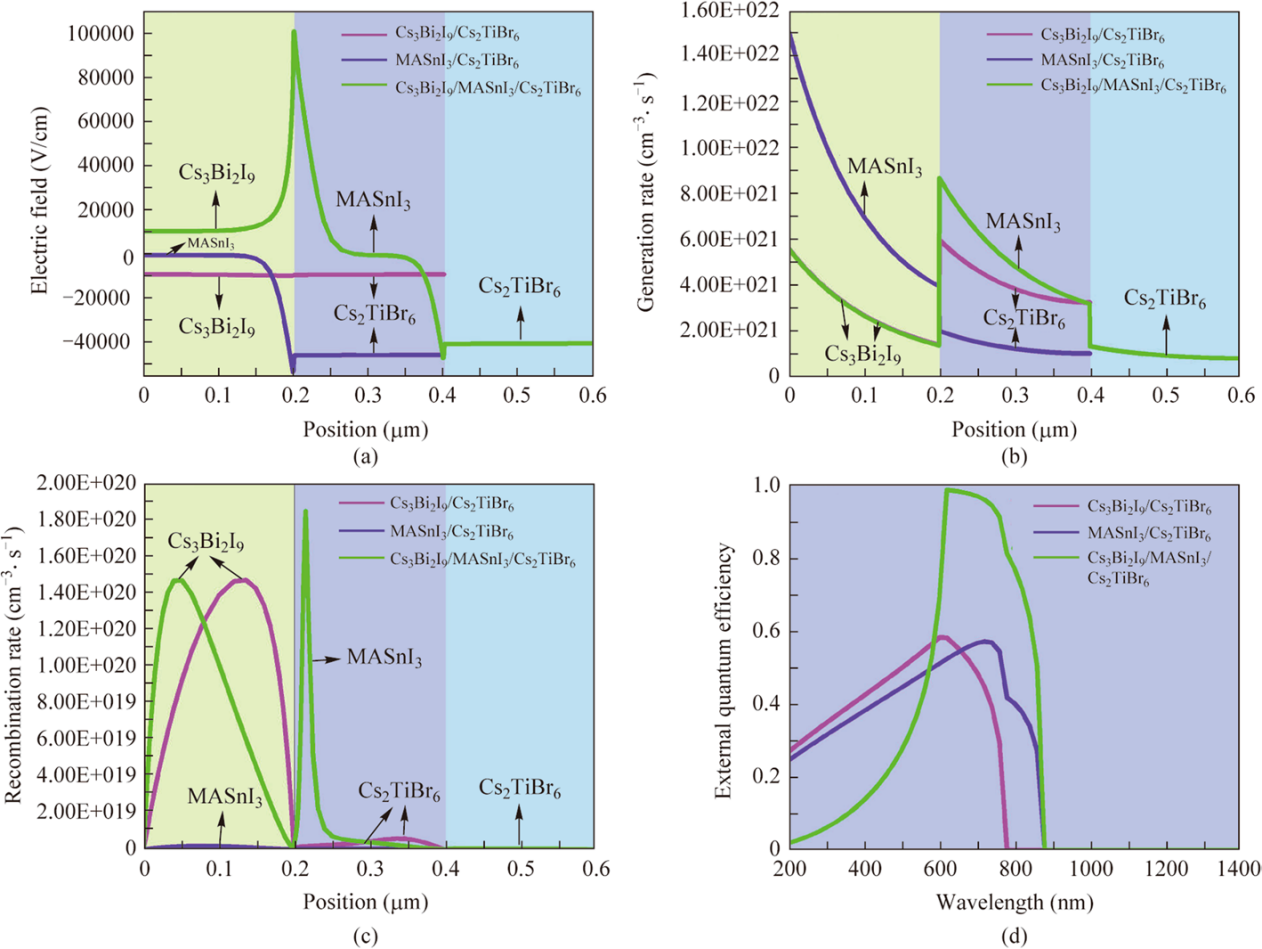
Diagrams of PSCs internal physical parameters with different absorber layers, **a** electric field distributions, **b** generation rate diagrams, **c** recombination rate diagrams, and **d** external quantum efficiency curves

The physical perception about the device performance of the proposed PSCs configuration with different absorber layers is revealed. The calculated electric field distribution, generation rate diagram, recombination rate diagram, and the external quantum efficiency (EQE) curves of PSCs are drawn as given in Fig. 3a − d, respectively. As can be observed in Fig. 3a, the electric field at the interface of p-type MASnI_3_/n-type Cs_2_TiBr_6_ with Cs_3_Bi_2_I_9_/MASnI_3_/Cs_2_TiBr_6_ absorber layer PSCs is slightly lower than MASnI_3_/Cs_2_TiBr_6_ absorber layer PSCs, this meaning that the thickness or doping of the absorber layer is still not adequately designed. Accordingly, more parameters need to be optimized to elevate device performance as will be done hereafter. Additionally, the contrary electric field distribution direction appears at the Cs_3_Bi_2_I_9_/MASnI_3_ interface. The contrary electric field behavior undeservedly influences the carrier collection, which heightens the recombination trend near the interface of Cs_3_Bi_2_I_9_/MASnI_3_ and results in the *V*_oc_ of the three absorber layer PSCs being slightly lower. This result can also be reflected in Fig. 3c. As presented by the generation rate-recombination rate diagrams in Fig. 3b and c, as observed highest carrier generation rate occurs at the surface or interface of the absorber layer in all device configurations (Fig. 3b). This may be interpreted as the presence of heterojunctions at the interface of the absorber layer. Moreover, the overall carrier generation rate of the MASnI_3_/Cs_2_TiBr_6_ and Cs_3_Bi_2_I_9_/MASnI_3_/Cs_2_TiBr_6_ absorber layer PSCs is higher than that of Cs_3_Bi_2_I_9_/Cs_2_TiBr_6_ absorber layer PSCs. This can be elucidated by the fact that the presence of MASnI_3_ with the smallest band gap and three absorber layers can absorb enough ultraviolet–visible light in both device architectures, implying the generation of higher carrier concentration and the *J*_sc_ and PCE sensibly improved. As can be observed in Fig. 3c, the low and even recombination rate occurs inside the MASnI_3_ and Cs_2_TiBr_6_ absorber layer. Nevertheless, the recombination losses significantly increase inside and near the top of the Cs_3_Bi_2_I_9_ absorber layer. This is mainly due to the overlarge band offset and the upward bending of the energy band at the interface of Cs_3_Bi_2_I_9_/Cs_2_TiBr_6_ and Cs_3_Bi_2_I_9_/MASnI_3_, respectively, as also illustrated in Fig. 2b and d. This also indicates that there are more defects inside and near the top of the Cs_3_Bi_2_I_9_ absorber layer. Therefore, the material parameters in the Cs_3_Bi_2_I_9_ absorber layer need to be additionally designed and optimized.

As depicted by the EQE response in Fig. 3d, the absorption spectrum of Cs_3_Bi_2_I_9_/Cs_2_TiBr_6_ absorber layer PSCs is around the wavelength of 770 nm. Nevertheless, in the case of the MASnI_3_/Cs_2_TiBr_6_ and Cs_3_Bi_2_I_9_/MASnI_3_/Cs_2_TiBr_6_ absorber layer PSCs, the MASnI_3_ absorber layer with a low band gap broadens the absorbance spectrum to the near-infrared wavelength range (around 870 nm), effectively facilitating the augmentation of photo generated electric and *J*_sc_ [31]. Additionally, it is worth noting that the Cs_3_Bi_2_I_9_/MASnI_3_/Cs_2_TiBr_6_ absorber layer configuration exhibits the highest EQE in the visible light range compared to the Cs_3_Bi_2_I_9_/Cs_2_TiBr_6_ and MASnI_3_/Cs_2_TiBr_6_ absorber layer configuration. The EQE can be described by

4 $$\text{EQE}=1240\times J_{\mathrm{sc}}/\lambda\times P_{\mathrm{in}},$$

where *λ* and *P*_in_ represent the total incident light power and light wavelength, respectively. It can be seen from Eq. (4) that the EQE in the visible light range is directly proportional to *J*_sc_. Therefore, the Cs_3_Bi_2_I_9_/MASnI_3_/Cs_2_TiBr_6_ three absorber layer configuration becomes efficient in terms of photon absorption. The *J*_sc_ and PCE of the three absorber layer PSCs can be enhanced remarkably.

### Three absorber layer PSCs with two different p-p-n and p-n–n heterojunctions

**Fig. 4:**
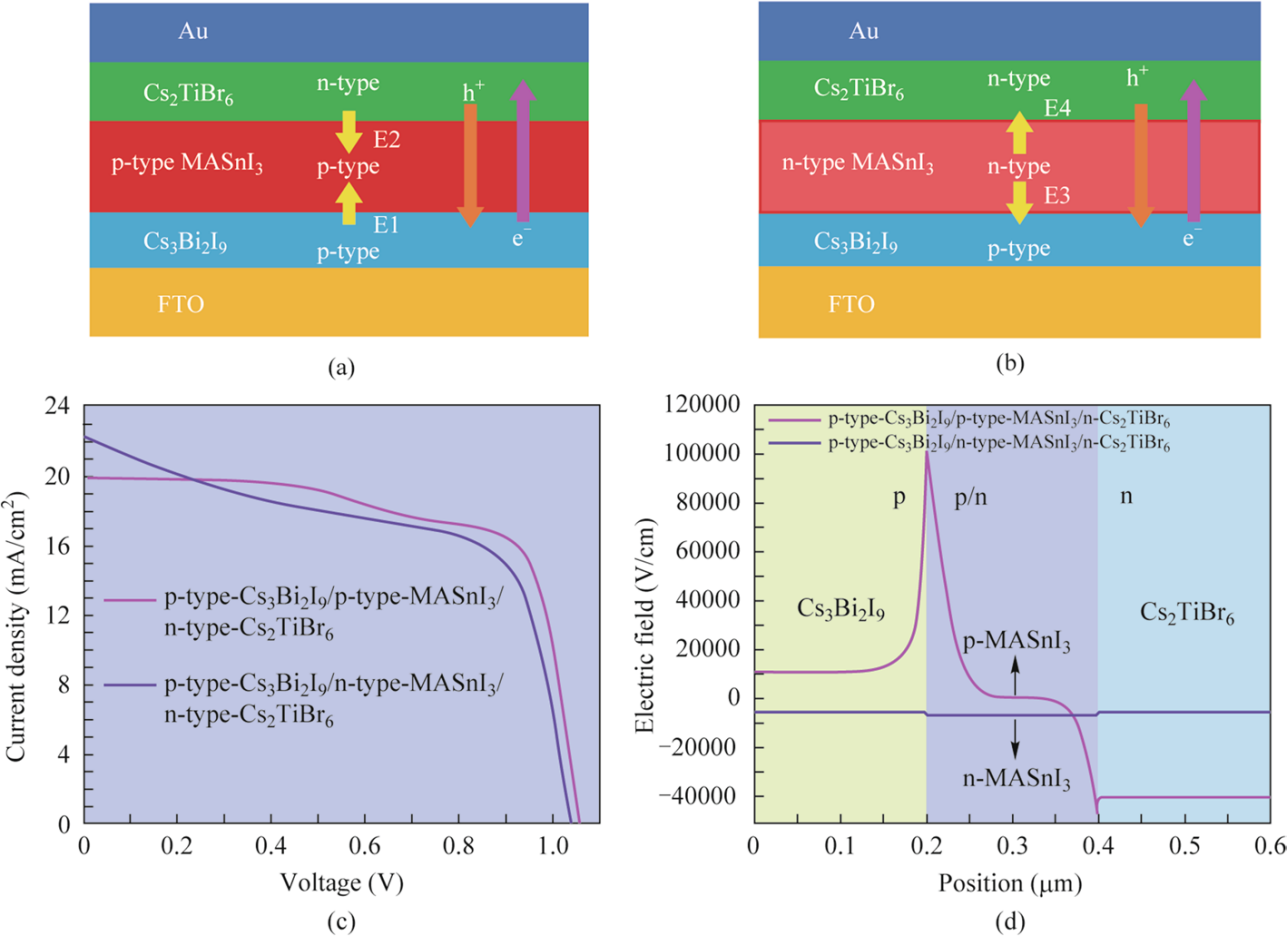
Device architecture diagrams of the three absorber layer PSCs with the **a** p-type-Cs_3_Bi_2_I_9_/p-type-MASnI_3_/n-type-Cs_2_TiBr_6_ and **b** p-type-Cs_3_Bi_2_I_9_/n-type-MASnI_3_/n-type-Cs_2_TiBr_6_ heterojunction structure, respectively. **c** *J* − *V* curves and **d** electric field distributions of the different heterojunction PSCs

In the PSC devices’ structure, different heterojunction structures will have specific effects on the spatial distribution and transmission of charge carriers [38]. Figure 4a and b depict the different device architecture diagrams of the proposed three absorber layer PSCs with the p-type-Cs_3_Bi_2_I_9_/p-type-MASnI_3_/n-type-Cs_2_TiBr_6_ and p-type-Cs_3_Bi_2_I_9_/n-type-MASnI_3_/n-type-Cs_2_TiBr_6_ heterojunction structure, respectively. Combining the PSCs *J* − *V* curves with different heterojunction structures in Fig. 4c and the electric field diagrams in Fig. 4d, it can be observed clearly in Fig. 4a that the strongest electric field (E1) in the upward direction is generated at the interface of p-type-Cs_3_Bi_2_I_9_/p-type-MASnI_3_, which is owing to the existence of the concentration difference at the interface. Furthermore, an intense electric field (E2) in the downward direction is generated at the interface of p-type-MASnI_3_/n-type-Cs_2_TiBr_6_ due to the presence of the p-n heterojunction. The electrons and holes in the device are separated and transported under the driving of the built-in electric field. The holes in Fig. 4a are transported from the n-type-Cs_2_TiBr_6_ absorber layer down to the FTO electrode. In virtue of the existence of E1 in the opposite direction of the hole transport, the transport and extraction of the hole are emasculated to a certain extent, thus impacting the *J*_sc_ of the devices. Nevertheless, in Fig. 4b, a weak electric field (E3) in the downward direction is generated at the interface of p-type-Cs_3_Bi_2_I_9_/n-type-MASnI_3_, and a weaker electric field (E4) in the upward direction is generated at the interface of n-type-MASnI_3_/n-type-Cs_2_TiBr_6_. Since the downward direction of E3 is slightly higher than the upward direction of E4, the remaining electric field in the downward direction will be more favorable for the transport and extraction of the hole compared to Fig. 4a. Therefore, it can be perceived from the performance parameters of devices with different heterojunction structures in Table 3 that the PSCs of p-type-Cs_3_Bi_2_I_9_/n-type-MASnI_3_/n-type-Cs_2_TiBr_6_ heterojunction structure has higher *J*_sc_ (22.3157 mA/cm^2^) than the value (19.9235 mA/cm^2^) from the PSCs of p-type-Cs_3_Bi_2_I_9_/p-type-MASnI_3_/n-type-Cs_2_TiBr_6_ heterostructure. It is also worth noting that the *V*_oc_ and FF of PSCs with p-type-Cs_3_Bi_2_I_9_/p-type-MASnI_3_/n-type-Cs_2_TiBr_6_ heterostructure are higher than those of PSCs with p-type-Cs_3_Bi_2_I_9_/n-type-MASnI_3_/n-type-Cs_2_TiBr_6_ heterostructure, which is perchance attributed to the diminution of series resistance and the strong electric field facilitates the separation of electrons and holes in the PSCs of the p-type-Cs_3_Bi_2_I_9_/p-type-MASnI_3_/n-type-Cs_2_TiBr_6_ heterojunction structure, effectively downgrading the recombination loss of carriers.

**Table 3:** Performance parameters of the devices with different heterojunction structures

Heterojunction structure	*V*_oc_ (V)	*J*_sc_ (mA/cm^2^)	FF (%)	PCE (%)
FTO/p-type-Cs_3_Bi_2_I_9_/p-type-MASnI_3_/n-type-Cs_2_TiBr_6_/Au	1.0569	19.9235	70.6818	14.8834
FTO/p-type-Cs_3_Bi_2_I_9_/n-type-MASnI_3_/n-type-Cs_2_TiBr_6_/Au	1.0373	22.3157	58.9315	13.6412

**Fig. 5:**
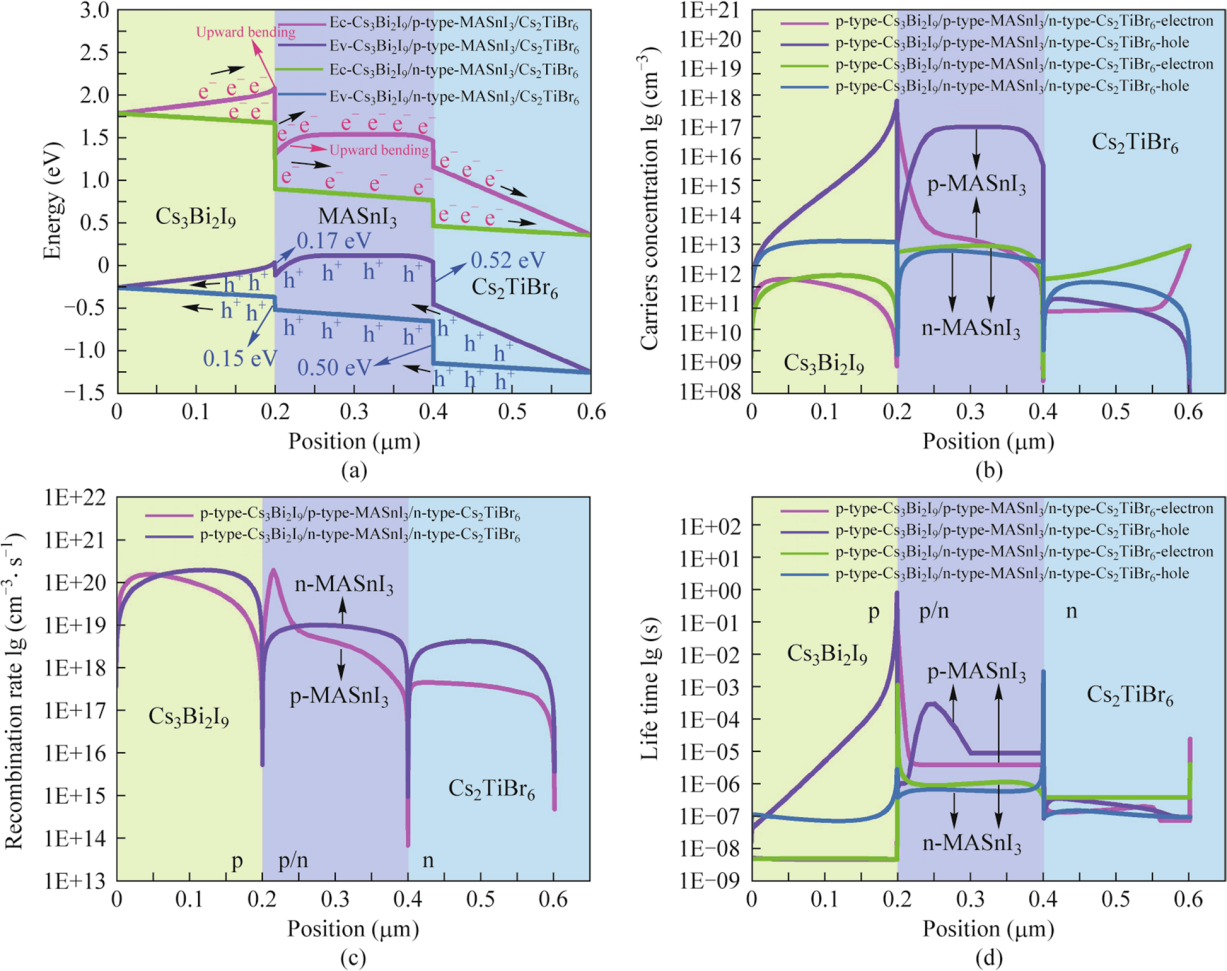
**a** Energy band diagrams, **b** carrier concentration distributions, **c** recombination rate curves, and **d** carrier life time curves of the different heterojunction PSCs

The energy band curves of the proposed PSCs with different heterojunction structures have been shown in Fig. 5a. In contrast with the energy band curve of the p-type-Cs_3_Bi_2_I_9_/p-type-MASnI_3_/n-type-Cs_2_TiBr_6_ heterojunction PSCs, there is no upward bending of the energy band at the interface of the absorber layer of the PSCs with p-type-Cs_3_Bi_2_I_9_/n-type-MASnI_3_/n-type-Cs_2_TiBr_6_ heterojunction structure. Consequently, the recombination of the carrier at the interface of the absorber layer with p-type-Cs_3_Bi_2_I_9_/n-type-MASnI_3_/n-type-Cs_2_TiBr_6_ heterojunction PSCs is lower, resulting in fewer defects. Both electrons and holes will be transported from the position with high energy values to the position with low energy values, which will be more conducive to the transport and extraction of carriers. This also demonstrates again the high *J*_sc_ of PSCs with p-type-Cs_3_Bi_2_I_9_/n-type-MASnI_3_/n-type-Cs_2_TiBr_6_ heterojunction structure. Figure 5b shows the variation curves of the carrier concentration values of PSCs with different heterojunction structures. We observe that there are hole and electron concentration differences at the interfaces of p-type-Cs_3_Bi_2_I_9_/p-type-MASnI_3_ and n-type-MASnI_3_/n-type-Cs_2_TiBr_6_, which is the reason for the presence of positive electric fields at the interface of these two absorber layers. Furthermore, it is worth mentioning that the concentration of the collective hole is higher than the electron concentration within both the p-type-Cs_3_Bi_2_I_9_ and p-type-MASnI_3_ absorber layers. However, the overall electron concentration is higher than the hole concentration in the n-type-MASnI_3_ and n-type-Cs_2_TiBr_6_ absorber layers. Correspondingly, the p-type perovskite absorber layer will be more favorable for the transmission of more electrons to the n-type perovskite absorber layer, and the n-type perovskite absorber layer will be more conducive to the transport of more holes to the p-type perovskite absorber layer. Accordingly, more holes and electrons are generated in the p-type and n-type perovskite absorber layers, respectively. As can be seen from the overall carrier concentration values in Fig. 5b, the p-type-Cs_3_Bi_2_I_9_/p-type-MASnI_3_/n-type-Cs_2_TiBr_6_ heterojunction produces higher electron and hole concentrations compared to the p-type-Cs_3_Bi_2_I_9_/n-type-MASnI_3_/n-type-Cs_2_TiBr_6_ heterojunction. Therefore, the high carrier concentration leads to high *V*_oc_ in p-type-Cs_3_Bi_2_I_9_/p-type-MASnI_3_/n-type-Cs_2_TiBr_6_ heterojunction PSCs. From the recombination rate curves of the PSCs with different heterojunction structures in Fig. 5c, it can be observed that the recombination rate in the p-type-Cs_3_Bi_2_I_9_/p-type-MASnI_3_/n-type-Cs_2_TiBr_6_ absorber layer is lower than that in the p-type-Cs_3_Bi_2_I_9_/n-type-MASnI_3_/n-type-Cs_2_TiBr_6_ absorber layer. Additionally, in the carrier life time curves of PSCs with different heterojunction structures shown in Fig. 5d, the carrier life time is only meaningful for minority carriers. The minority carriers are electrons in p-type perovskite semiconductors, and the minority carriers are holes in n-type perovskite semiconductors. Then it is evident that the carrier life time in the p-type MASnI_3_ absorber layer is higher than that in the n-type MASnI_3_ absorber layer, and the n-type-Cs_2_TiBr_6_ absorber layer in the p-type-Cs_3_Bi_2_I_9_/p-type-MASnI_3_/n-type-Cs_2_TiBr_6_ heterojunction PSCs has a higher carrier life time compared to the p-type-Cs_3_Bi_2_I_9_/n-type-MASnI_3_/n-type-Cs_2_TiBr_6_ heterojunction PSCs. Combined with the above analysis, as can be concluded that PSCs with p-type-Cs_3_Bi_2_I_9_/p-type-MASnI_3_/n-type-Cs_2_TiBr_6_ heterojunction structure have higher *V*_oc_, FF as well as PCE due to less carrier recombination and higher carrier life time inside the absorber layers. The following are further studies on the PSCs with p-p-n heterojunction structure.

### Three absorber layer PSCs with different double perovskite materials

**Fig. 6:**
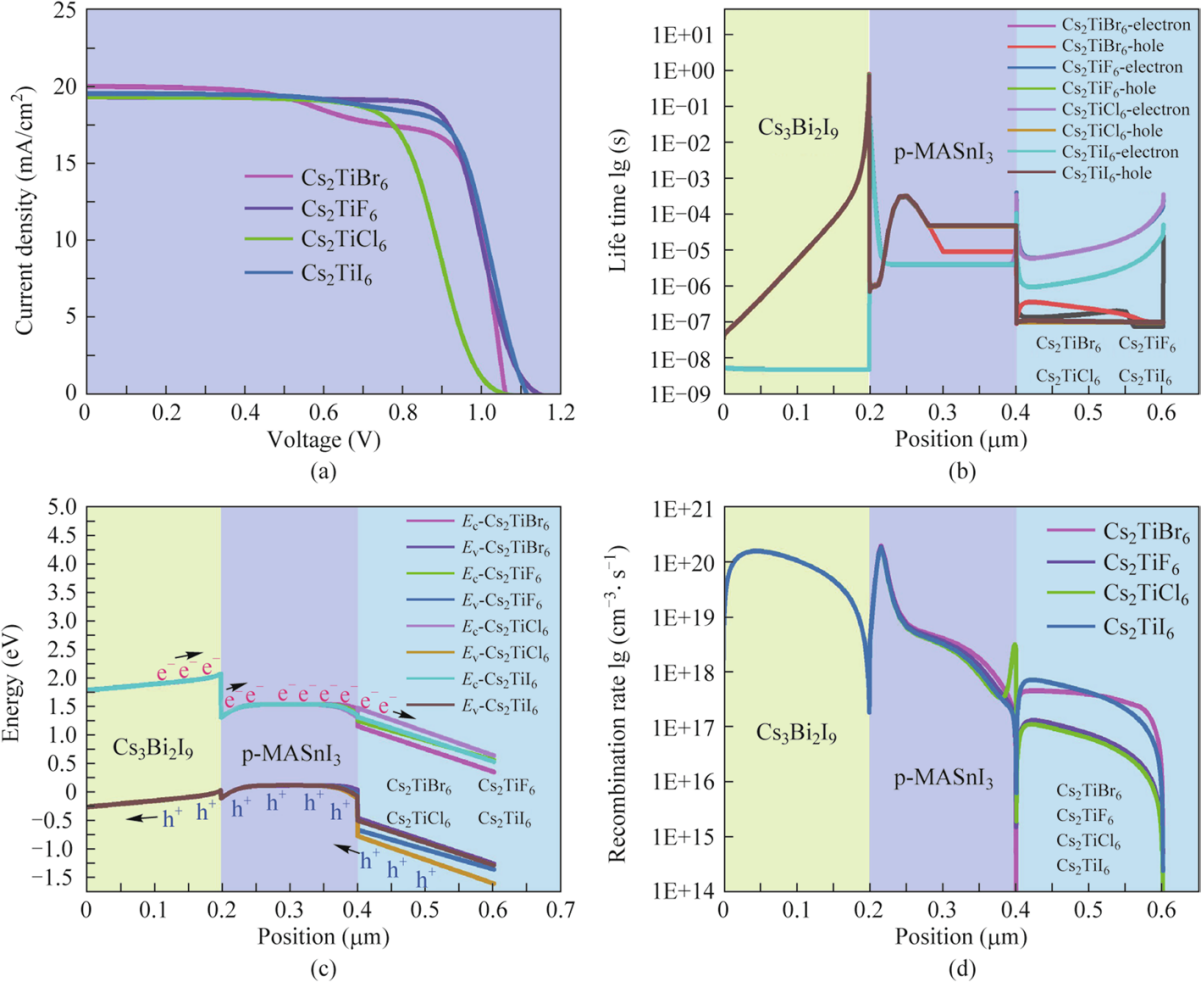
**a** *J* − *V* curves, **b** carrier life time curves, **c** energy band diagrams and **d** recombination rate curves of the three absorber layer PSCs with various double perovskite materials

The double perovskite materials of the proposed three absorber layer PSCs play a crucial role in transporting electrons from the perovskite layer to the metal electrode [39]. Since Cs_3_Bi_2_I_9_ and MASnI_3_ are already optimal triple perovskite absorber layer materials and single perovskite absorber layer materials in our simulated three absorber layer PSCs. Accordingly, to simplify the content of our manuscript, the impact of utilizing various double perovskite materials (Cs_2_TiBr_6_, Cs_2_TiF_6_, Cs_2_TiCl_6_, Cs_2_TiI_6_) on the photoelectric performance of the proposed three absorber layer PSC is merely investigated in this section. The details of double perovskite materials parameters used in our simulations were summarized from cognate literature as displayed in Table 4 [20, 36, 39, 40]. The *J* − *V* curves and performance parameters of three absorber layer PSCs with various double perovskite materials are presented in Fig. 6a and Table 5, respectively. The detections in Fig. 6a and Table 5 display the highest *J*_sc_ in the three absorber layer PSCs with double Cs_2_TiBr_6_ materials. Combining the carrier life time curves and energy band alignment of PSCs with various double perovskite materials in Fig. 6b and c, respectively. It can be interpreted clearly that the highest carrier (hole) life time occurs in the Cs_2_TiBr_6_ absorber layer and more holes can be transported and extracted into the conductive substrate. Nevertheless, the maximum *V*_oc_ and FF occur in the PSCs with double Cs_2_TiF_6_ materials, which is attributed to the ideal band alignment at the interface of p-type-MASnI_3_/n-type-Cs_2_TiF_6_ and lower carrier recombination loss in the Cs_2_TiF_6_ and MASnI_3_ absorber layers from Fig. 6d. To sum up, the maximum *V*_oc_ and FF give rise to the highest PCE of 16.1736% when the Cs_2_TiF_6_ is regarded as the double perovskite material of the three absorber layer PSCs.

**Table 4:** Materials parameters of the double perovskite

Parameters	Cs_2_TiBr_6_	Cs_2_TiF_6_	Cs_2_TiCl_6_	Cs_2_TiI_6_
Thickness (µm)	0.2	0.2	0.2	0.2
Relative permittivity *ε*_r_	10	18	19	18
Electron affinity *χ* (eV)	4.47	4.3	4	4.2
Band gap *E*_g_ (eV)	1.6	1.9	2.23	1.8
Effective conduction band density *N*_c_ (cm^−3^)	1 × 10^19^	1 × 10^19^	1 × 10^19^	1 × 10^19^
Effective valence band density *N*_v_ (cm^−3^)	1 × 10^19^	1 × 10^19^	1 × 10^19^	1 × 10^18^
Electron mobility *μ*_n_ (cm^2^/(V⋅s))	44	4.4	4.4	4.4
Hole mobility *μ*_p_ (cm^2^/(V⋅s))	2.5	2.5	2.5	2.5
Acceptor density *N*_A_/cm^−3^	0	1 × 10^19^	1 × 10^19^	1 × 10^19^
Donor density *N*_D_/cm^−3^	1 × 10^13^	1 × 10^19^	1 × 10^19^	1 × 10^19^
Defect density *N*_t_/cm^−3^	1 × 10^14^	1 × 10^14^	1 × 10^14^	1 × 10^14^

**Table 5:** Performance parameters of three absorber layer PSCs with various double perovskite materials

Parameters	*V*_oc_ (V)	*J*_sc_ (mA/cm^2^)	FF (%)	PCE (%)
Cs_2_TiBr_6_	1.0569	19.9235	70.6818	14.8834
Cs_2_TiF_6_	1.1468	19.2192	73.3815	16.1736
Cs_2_TiCl_6_	1.0621	19.2255	66.2122	13.5203
Cs_2_TiI_6_	1.1118	19.4532	72.7802	15.7416

### Effect of Cs_3_Bi_2_I_9_/MASnI_3_/Cs_2_TiF_6_ absorber layer thicknesses on the performance of the three absorber layer PSCs

**Fig. 7:**
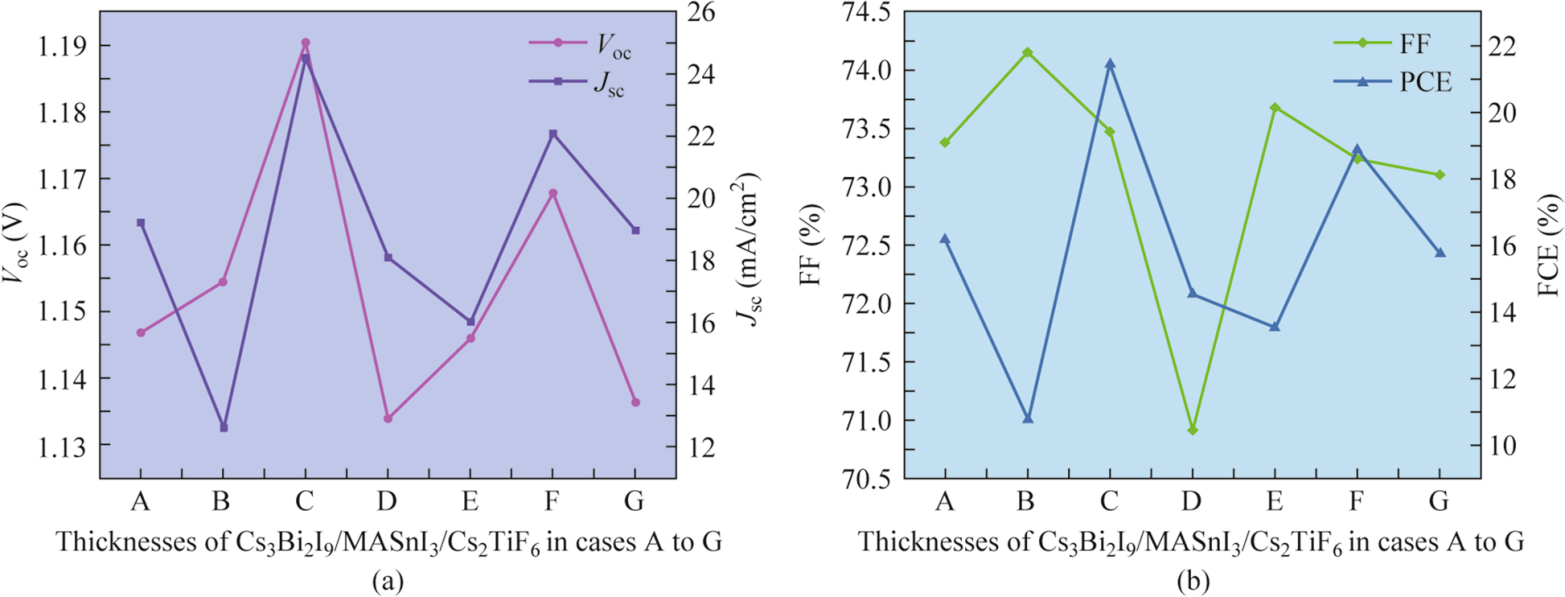
Effect of Cs_3_Bi_2_I_9_, MASnI_3_ and Cs_2_TiF_6_ absorber layer thicknesses variation on **a** *V*_oc_ and *J*_sc_, **b** FF and PCE of the three absorber layer PSCs

The thicknesses and photoelectric parameters of the absorber layer have become decisive factors affecting the performance of the PSCs [41]. We have devised seven combinations of the Cs_3_Bi_2_I_9_/MASnI_3_/Cs_2_TiF_6_ absorber layer thicknesses (noted from A to G) while maintaining the overall thickness of the three absorber layers invariant at 0.6 µm. Figure 7a and b and Table 6 display the performance parameters of the device with different Cs_3_Bi_2_I_9_/MASnI_3_/Cs_2_TiF_6_ absorber layer thicknesses. The excessive absorber layer thickness may eventuate an evident increment in the carrier recombination and series resistance, whereas the thin absorber layer thickness perchance induces less shunt resistance [42]. Among the simulated results with various three absorber layer thicknesses. We can observe case C (0.1/0.4/0.1 µm) in which the thickest MASnI_3_ absorber layer (400 nm) bespeaks the highest PCE of 21.4530% corresponds to an evident enhancement compared to the PCE of case A (0.2/0.2/0.2 µm (16.1736%)). On the one hand, this enhancement in PCE is due to the increasing thickness of the MASnI_3_ absorber layer, which has the narrowest band gap (1.41 eV) and can hasten a wider range of photon absorption, thus more photogenerated carriers are generated. On the other hand, the Cs_3_Bi_2_I_9_ absorber layer can be comprehensively covered with the increasing of MASnI_3_ thickness, effectively eschewing the direct contact between the Cs_2_TiF_6_ absorber layer and the Cs_3_Bi_2_I_9_ absorber layer. Consequently, not only the series resistance is possibly reduced, but also the leakage is effectively avoided. However, when the thickness of MASnI_3_ absorber layer is less than 0.4 µm, the *V*_oc_ and *J*_sc_ shall gradually dilute with the increasing of the Cs_3_Bi_2_I_9_ or Cs_2_TiF_6_ absorber layer thickness. This is attributed to the excessive Cs_3_Bi_2_I_9_ or Cs_2_TiF_6_ absorber layer thickness with a wide band gap eventuating more defects and carrier recombination losses inside and at the interface of the absorber layer, and the carriers need to be diffused over a longer distance before reaching the corresponding electrodes.

**Table 6:** Device performance parameters for the thicknesses of three absorber layers in cases A to G

Case thickness of Cs_3_Bi_2_I_9_/MASnI_3_/Cs_2_TiF_6_ (µm)	*V*_oc_ (V)	*J*_sc_ (mA/cm^2^)	FF (%)	PCE (%)
A 0.2/0.2/0.2	1.1468	19.2192	73.3815	16.1736
B 0.4/0.1/0.1	1.1544	12.5742	74.1557	10.7642
C 0.1/0.4/0.1	1.1904	24.5295	73.4706	21.4530
D 0.1/0.1/0.4	1.1339	18.0953	70.9085	14.5492
E 0.3/0.15/0.15	1.1460	16.0215	73.6804	13.5283
F 0.15/0.3/0.15	1.1678	22.0727	73.2437	18.8801
G 0.15/0.15/0.3	1.1364	18.9724	73.1056	15.7618

### Effect of Cs_3_Bi_2_I_9_ acceptor doping concentration on the performance of the three absorber layer PSCs

**Fig. 8:**
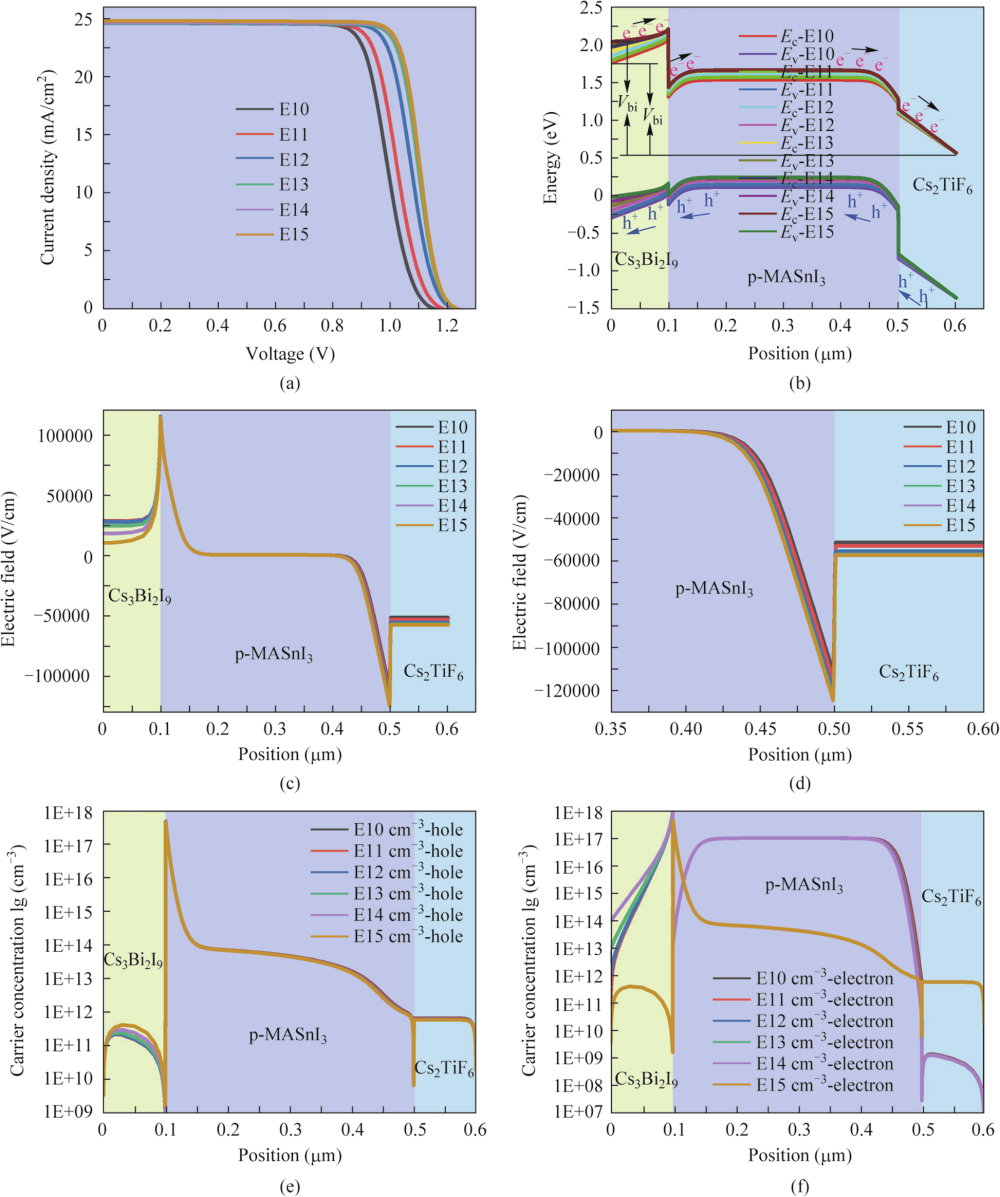
Effect of Cs_3_Bi_2_I_9_ acceptor doping concentration variation on **a** *J* − *V* curves, **b** energy band diagrams, **c** and **d** electric field, **e** hole concentration and **f** electron concentration curves of the three absorber layer PSCs

The doping concentration of the absorber layer has become a crucial factor for regulating the photoelectronic properties of PSCs, which directly affects the generation and transport of photogenerated carriers, hence involving the performance of the device [43, 44]. From a practical point of view, we took Cs_3_Bi_2_I_9_ acceptor doping concentration between E10 cm^−3^ and E15 cm^−3^ to maintain the rationality of device parameters. The impacts of Cs_3_Bi_2_I_9_ acceptor doping concentration on *J* − *V* curves and energy band distributions are plotted in Fig. 8a and b, respectively. The performance parameters of three absorber layer PSCs with various Cs_3_Bi_2_I_9_ acceptor doping concentrations are displayed in Table 7. Figure 8a and Table 7 display that the *J*_sc_ and *V*_oc_ improve smoothly with the Cs_3_Bi_2_I_9_ acceptor doping concentrations increases. The bending of the energy band increases in the Cs_3_Bi_2_I_9_ absorber layer giving rise to the enhancement of *V*_bi_, as seen in Fig. 8b, and the larger *V*_bi_ can effectively facilitate the separation and migration of carriers toward corresponding electrodes, thus making for a higher *V*_oc_. Additionally, this result can be interpreted from the electric field diagrams in Fig. 8c and d. It can be noticed definitely that the strongest electric field is generated at the interface of MASnI_3_/Cs_2_TiF_6_ when the Cs_3_Bi_2_I_9_ acceptor doping concentration is increased to E15 cm^−3^, which facilitates the transport and extraction of carriers. The *J*_sc_ and FF are all improved marginally with the increased doping concentration of the Cs_3_Bi_2_I_9_ absorber layer, which can be elucidated by

**Table 7:** Performance parameters of three absorber layer PSCs with various Cs_3_Bi_2_I_9_ acceptor doping concentrations

Cs_3_Bi_2_I_9_ doping concentration (cm^−3^)	*V*_oc_ (V)	*J*_sc_ (mA/cm^2^)	FF (%)	PCE (%)
E10	1.1596	24.5276	73.3815	20.6603
E11	1.1904	24.5295	73.4706	21.4530
E12	1.2271	24.5357	75.3780	22.6952
E13	1.2380	24.5580	77.1516	23.4560
E14	1.2395	24.6131	77.6182	23.6789
E15	1.2397	24.7322	77.6191	23.7982

5 $$N_{\mathrm A}=2\varepsilon\varepsilon_0/(qW^2).$$

In Eq. (5), *W* and *N*_A_ represent depletion region width and acceptor doping concentration, respectively. With increased Cs_3_Bi_2_I_9_ acceptor doping concentration, the depletion layer width narrows as Cs_3_Bi_2_I_9_ acceptor doping concentration increases, lowering the impediment to majority carrier movement and thus improving the FF. In the case of *J*_sc_, the collection of photogenerated carriers may be diminished with the increases in neutral region width, making for higher bulk recombination. In that way, the *J*_sc_ may be affected somewhat. Nevertheless, the *J*_sc_ shows a slow growth inclination. This is attributed to the increased Cs_3_Bi_2_I_9_ doping concentration resulting in the energy band bending of the Cs_3_Bi_2_I_9_ absorber layer in Fig. 8b, thus facilitating that the carriers can be efficiently transported. Additionally, it can be also seen from the hole concentration and electron concentration curves in Fig. 8e and f that both hole concentration and electron concentration are all significantly increased in the Cs_3_Bi_2_I_9_ absorber layer and Cs_2_TiF_6_ absorber layer, respectively, with the increasing of Cs_3_Bi_2_I_9_ acceptor doping concentrations. Then more electrons and holes can be efficiently transported and collected to the respective electrodes. Consequently, it can be concluded that when the doping concentration is extracted E15 cm^−3^ in the Cs_3_Bi_2_I_9_ absorber layer shall yield 23.7982% high-performance photoelectric devices.

### Effect of MASnI_3_ acceptor doping concentration on the performance of the three absorber layer PSCs

**Fig. 9:**
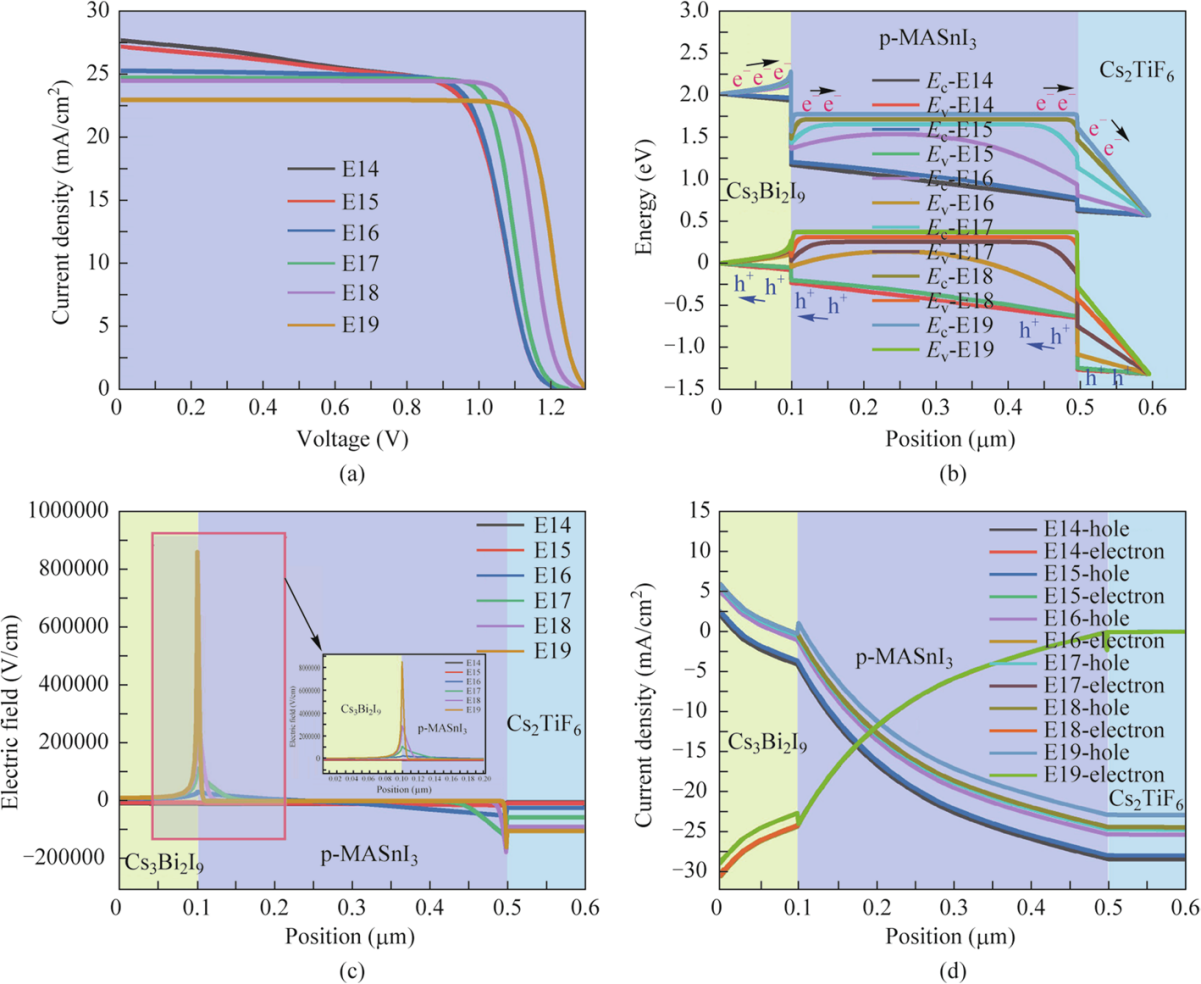
Effect of MASnI_3_ acceptor doping concentration variation on **a** *J* − *V* curves, **b** energy band diagrams, **c** electric field and **d** carriers’ current density curves of the three absorber layer PSCs

Figure 9a and b depict the alterations in *J* − *V* curves and energy band distributions of the three absorber layer PSCs as a function of different acceptor doping concentrations (E14 cm^−3^ − E19 cm^−3^) in the MASnI_3_ absorber layer, respectively. The relevant performance parameters of PSCs are plotted in Table 8. As can be observed in Fig. 9a and Table 8, the *V*_oc_ and FF improve continuously with increased doping concentrations of the MASnI_3_ absorber layer. This can be also interpreted by Eq. (5), the width of the depletion layer in the MASnI_3_/Cs_2_TiF_6_ heterojunction narrows as MASnI_3_ acceptor doping concentration increases, lowering the impediment to majority carrier movement and thus the existence of majority carriers (holes) in the p-type MASnI_3_ absorber layer heightening the FF. From energy band curves in Fig. 9b, when the doping concentration exceeds E16 cm^−3^ in the MASnI_3_ absorber layer, two lesser band offsets in the conduction band and valence band at the interface of Cs_3_Bi_2_I_9_/ MASnI_3_ can be conducive to the improvement of *V*_oc_. Additionally, the stronger electric field at the interface of Cs_3_Bi_2_I_9_/ MASnI_3_ and MASnI_3_/Cs_2_TiF_6_ when the doping concentration of MASnI_3_ absorber layer exceeds E17 cm^−3^, as displayed in Fig. 9c, further facilitating the separation and transport of carriers, and thereby enhancing the *V*_oc_ of the device significantly. Nevertheless, the *J*_sc_ diminishes gradually as the doping concentration of the MASnI_3_ absorber layer increases, this is due to the collection of photogenerated carriers being impeded as the increases in neutral region width under the high doping concentration, inducing a gradual increase in bulk recombination. As can be seen obviously from the current density curves of the carriers in Fig. 9d, the hole current density is also diminished gradually as the MASnI_3_ absorber layer doping concentration increases, which directly affects the *J*_sc_ of the device. Therefore, the excessively high doping concentration of the MASnI_3_ absorber layer should not be selected. Overall, based on the above analysis, the high PCE of 25.0713% can be successfully obtained when dominating the MASnI_3_ absorber layer doping concentration at about E18 cm^−3^.

**Table 8:** Performance parameters of three absorber layer PSCs with various MASnI_3_ acceptor doping concentrations

MASnI_3_ doping concentration (cm^−3^)	*V*_oc_ (V)	*J*_sc_ (mA/cm^2^)	FF (%)	PCE (%)
E14	1.2246	27.7240	65.0079	22.0709
E15	1.2246	27.2014	66.2816	22.0792
E16	1.2250	25.2849	72.7471	22.5332
E17	1.2397	24.7322	77.6191	23.7982
E18	1.2793	24.4813	80.0519	25.0713
E19	1.3037	22.9641	81.4962	24.3981

### Effect of Cs_2_TiF_6_ donor doping concentration on the performance of the three absorber layer PSCs

**Fig. 10:**
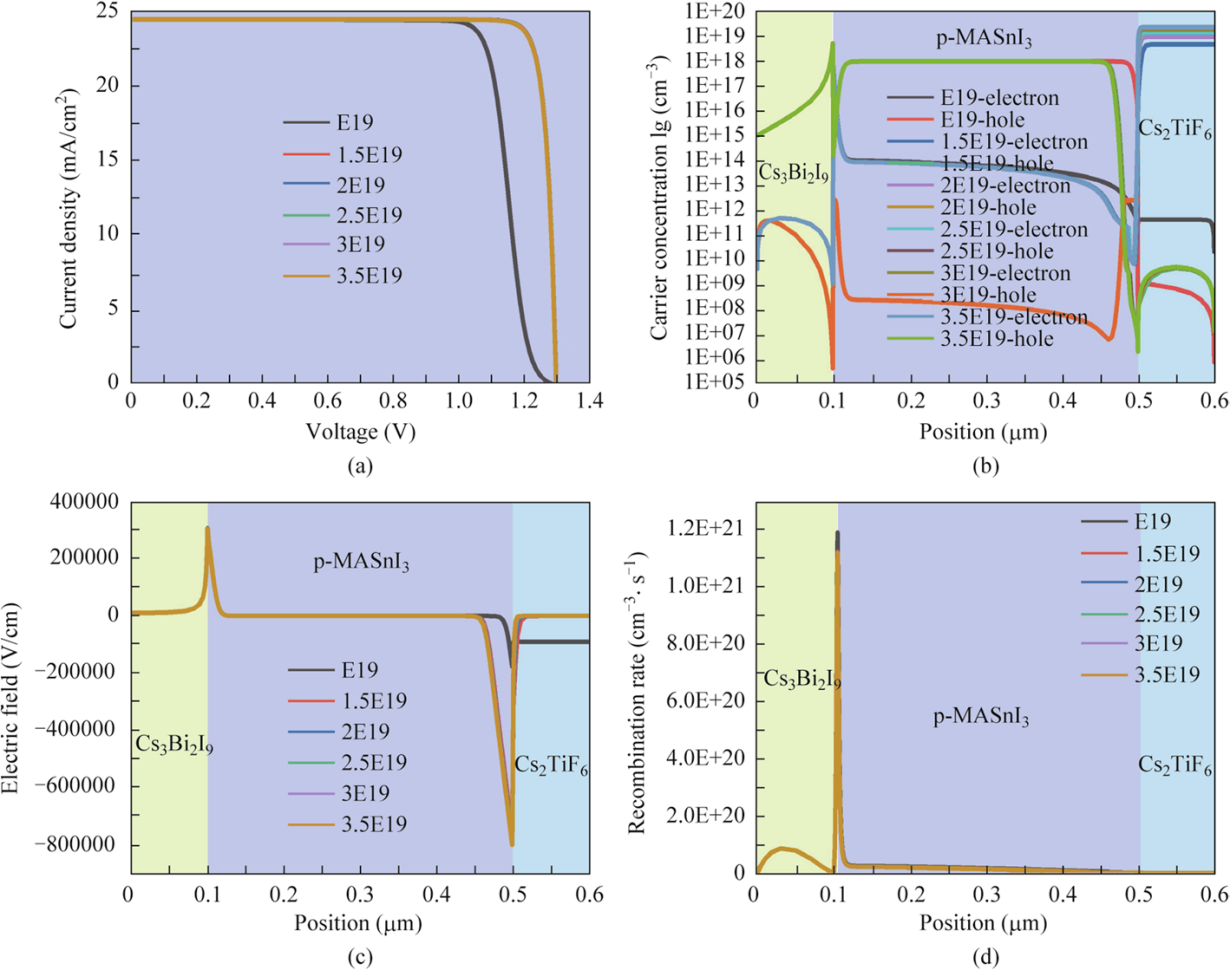
Effect of Cs_2_TiF_6_ donor doping concentration variation on **a** *J* − *V* curves, **b** carrier concentration distributions, **c** electric field, and **d** recombination rate curves of the three absorber layer PSCs

The donor doping concentration is also a momentous parameter, which directly impacts the photoelectric performance of the device. Therefore, an exploration of the impact of the Cs_2_TiF_6_ donor doping concentration on the performance of the three absorber layer PSCs was conducted. Figure 10a and Table 9 present the *J*–*V* curves and performance parameters of the three absorber layer PSCs as a function of the donor doping concentration of the Cs_2_TiF_6_ absorber layer variations from E19 cm^−3^ to 3.5E19 cm^−3^. The *V*_oc_, FF, and PCE improve with the increases of Cs_2_TiF_6_ doping concentration from E19 cm^−3^ to 1.5E19 cm^−3^ and then decline marginally as the Cs_2_TiF_6_ absorber layer doping concentration increases from 1.5E19 cm^−3^ to 3.5E19 cm^−3^. However, the *J*_sc_ changes barely with the increasing doping concentration of the Cs_2_TiF_6_ absorber layer. The PCE reaches the maximum when the Cs_2_TiF_6_ absorber layer doping concentration is 1.5E19 cm^−3^. From the carrier concentration curves (Fig. 10b) and electric field distribution (Fig. 10c), respectively, it can be visibly seen that the electron concentration inside the Cs_2_TiF_6_ absorber layer and the electric field strength at the interface of MASnI_3_/Cs_2_TiF_6_ all increase gradually with the increase of Cs_2_TiF_6_ doping concentration. Additionally, there are fewer carriers’ recombination when the doping concentration of the Cs_2_TiF_6_ absorber layer surpasses E19 cm^−3^ (as shown in Fig. 10d). Accordingly, fewer carriers will be trapped by the defect, and more carriers will be efficiently separated and transported to the corresponding electrode. In summary analysis, it can be determined explicitly that a moderate donor doping concentration (1.5E19 cm^−3^) in the Cs_2_TiF_6_ absorber layer will yield 28.6193% optimal-performance PSCs devices.

**Table 9:** Performance parameters of three absorber layer PSCs with various Cs_2_TiBr_6_ donor doping concentrations

Cs_2_TiBr_6_ doping concentration (cm^−3^)	*V*_oc_ (V)	*J*_sc_ (mA/cm^2^)	FF (%)	PCE (%)
1E19	1.2793	24.4813	80.0519	25.0713
1.5E19	1.3012	24.4844	89.8337	28.6193
2E19	1.3011	24.4844	89.7787	28.6008
2.5E19	1.3011	24.4845	89.7292	28.5842
3E19	1.3010	24.4846	89.6886	28.5705
3.5E19	1.3010	24.4846	89.6508	28.5577

## Conclusions

A numerical simulation investigation on the novel design of CTLs-free inverted PSCs with triple Cs_3_Bi_2_I_9_/single MASnI_3_/double Cs_2_TiBr_6_ absorber layers has been unfolded in this work to pick the optimal conditions by employing wxAMPS software. The simulation demonstrates that the three absorber layer architecture of PSCs is more conducive to the transport and extraction of more holes from the valence band to the electrode and has better photoelectric characteristics than that of the two absorber layers architecture of PSCs, and the higher PCE (14.8834%) of the simulated device with Cs_3_Bi_2_I_9_/MASnI_3_/Cs_2_TiBr_6_ heterojunction has been obtained. Meanwhile, the simulation results illuminate that the electrical and photophysical properties of the device with p-type-Cs_3_Bi_2_I_9_/p-type-MASnI_3_/n-type-Cs_2_TiBr_6_ heterojunction architecture (p-p-n) is superior to the one with p-type-Cs_3_Bi_2_I_9_/n-type-MASnI_3_/n-type-Cs_2_TiBr_6_ heterojunction architecture (p-n–n) due to less carrier recombination and higher carrier life time inside the absorber layers. The oxidation of Sn^2+^ in MASnI_3_ materials will result in p-type doping of the MASnI_3_ layer. Therefore, we also believe that the p-type MASnI_3_ is generally caused by the quantitative oxidation of Sn^2+^ may have a positive impact on devices of Sn-based perovskites under our device structure (p-p-n). Additionally, after optimizing the parameters of each absorber layer, we obtained the larger *V*_oc_ and FF give rise to the better PCE of 16.1736% when the Cs_2_TiF_6_ is regarded as the double perovskite material of the three absorber layer PSCs. Varying the thickness of each absorber layer, it was revealed that the thicknesses of absorber layers have a remarkable influence on the *J*_sc_ of the device, and the thicker MASnI_3_ absorber layer can obtain an excellent PCE of 21.4530%. Lastly, the highest-performance (28.6193%) photoelectric devices can be created with the optimized doping density of around E15 cm^3^, E18 cm^3^, and 1.5E19 cm^3^ in the Cs_3_Bi_2_I_9_ absorber layer, MASnI_3_ absorber layer, and Cs_2_TiBr_6_ absorber layer, respectively. This work not only simplifies the fabrication process of the devices but also allows us to better understand the spatial distribution of internal carriers and charge transport mechanism of the different structure devices. What's more, this work will also tender theoretical guidance for the large-scale fabrication of high-performance CTLs-free inverted PSCs with multi-absorber layers.

## Data Availability

The data that support the findings of this study are available from the corresponding author, upon reasonable request.
